# Musical training refines audiovisual integration but does not influence temporal recalibration

**DOI:** 10.1038/s41598-022-19665-9

**Published:** 2022-09-12

**Authors:** Matthew O’Donohue, Philippe Lacherez, Naohide Yamamoto

**Affiliations:** School of Psychology and Counselling, Queensland University of Technology (QUT), Kelvin Grove, QLD 4059 Australia

**Keywords:** Cognitive neuroscience, Neuroscience, Psychology, Human behaviour

## Abstract

When the brain is exposed to a temporal asynchrony between the senses, it will shift its perception of simultaneity towards the previously experienced asynchrony (temporal recalibration). It is unknown whether recalibration depends on how accurately an individual integrates multisensory cues or on experiences they have had over their lifespan. Hence, we assessed whether musical training modulated audiovisual temporal recalibration. Musicians (*n* = 20) and non-musicians (*n* = 18) made simultaneity judgements to flash-tone stimuli before and after adaptation to asynchronous (± 200 ms) flash-tone stimuli. We analysed these judgements via an observer model that described the left and right boundaries of the temporal integration window (decisional criteria) and the amount of sensory noise that affected these judgements. Musicians’ boundaries were narrower (closer to true simultaneity) than non-musicians’, indicating stricter criteria for temporal integration, and they also exhibited enhanced sensory precision. However, while both musicians and non-musicians experienced cumulative and rapid recalibration, these recalibration effects did not differ between the groups. Unexpectedly, cumulative recalibration was caused by auditory-leading but not visual-leading adaptation. Overall, these findings suggest that the precision with which observers perceptually integrate audiovisual temporal cues does not predict their susceptibility to recalibration.

## Introduction

Integration of sensory cues is fundamental to achieving robust and coherent perceptual experiences. For example, monitoring speech is an important everyday task that requires observers to integrate auditory (speech sound) and visual (facial movement) cues. Multisensory experiences such as these are affected by various sources of noise inherent in the environment. For example, it is well known that sound travels more slowly than light through air, yet when physical distance is negligible, sound activates the auditory cortex faster than light activates the visual cortex^1–3^. Accordingly, the past two decades of research have shown that the brain flexibly adjusts its perception of spatial and temporal alignment via a compensatory mechanism called recalibration^4–6^. Recalibration effects that last for at least 1 min are produced by several minutes of exposure (i.e., adaptation) to spatially or temporally misaligned stimuli (cumulative recalibration), but transient recalibration effects can also be seen between individual stimulus presentations (rapid or inter-trial recalibration)^7,8^. Given the intuitive assumption that recalibration should only occur when it is relatively likely that the sensory cues were causally associated, multisensory integration and recalibration seem to be closely related. However, we know little about the extent to which recalibration depends on integration or on experiences that may have modulated how we process multisensory signals.

Van der Burg et al.^9^ first demonstrated rapid temporal recalibration and found that there was a significant negative correlation between audiovisual temporal acuity and the magnitude of recalibration. That is, observers who perceived simultaneity between flashes and tones over a narrower range of stimulus onset asynchronies (SOAs) tended to experience less recalibration, supporting the notion that the brain should only compensate for sensory misalignment when the cues are perceived to belong to the same event. This finding was corroborated by some later studies^10–12^ and a similar association has also been found in the spatial domain where an observer’s tendency to integrate predicts their susceptibility to rapid recalibration^13,14^ but not cumulative recalibration^13,15^. However, more recent work has suggested that in the temporal domain, the magnitude of integration does not predict the magnitude of recalibration. Noel et al.^10^ showed that previous correlations between integration and rapid recalibration were probably driven by age, whereby observers experience less recalibration as they age. Van der Burg et al.^16^ found that rapid recalibration was driven by the physical rather than the *perceived* temporal order of the preceding audiovisual stimulus. This may suggest that temporal recalibration is not predicted by how liberally individuals integrate sensory cues and is instead determined by the objective temporal disparity between sensory cues. In summary, while rapid *spatial* recalibration appears to be at least partly driven by multisensory integration, it is unclear whether *temporal* recalibration is related to the precision with which individuals integrate multisensory cues. Thus, it would be informative to assess whether observers with multisensory expertise would experience temporal recalibration differently from observers without expertise.

We used musicians and non-musicians to explore this. While other forms of expertise, such as bilingualism^17^ and video gaming experience^18^, improve multisensory temporal integration, musical training is the most prominent example of expertise in multisensory timing literature^19^. Musical training involves precise integration of motor actions with sounds, visual cues (e.g., a piano key being pressed), and somatosensory feedback^20^. Accordingly, a small but growing body of research indicates that musicians have more refined temporal integration for audiotactile and audiovisual stimuli. Lee and Noppeney^21,22^ showed that pianists integrate musical stimuli, and possibly speech, over a narrower range of SOAs than non-musicians. Functional magnetic resonance imaging (fMRI) data in this study also found that musicians had increased neural responses to asynchronous musical cues in various brain regions, such as the cerebellum. Lu et al.^23^ provided behavioural evidence that musicians exhibit refined audiovisual integration in a domain-general context (i.e., for simple stimuli unrelated to music or speech), as when judging the simultaneity of flashes and tones. Lu et al. also found, using magnetoencephalography (MEG), that musicians exhibited greater neural activity in various temporal regions of the brain in response to synchronous stimuli, and greater activity in the cerebellum in response to asynchronous stimuli. Later studies found that musicians demonstrate greater multisensory integration as measured by audiotactile reaction times^24^, and are less susceptible to the sound-induced flash illusion^25^ and its audiotactile equivalent^26^.

Most recently, Jicol et al.^27^ conducted a temporal recalibration experiment with drummers, non-drummer musicians, and non-musicians. As expected, both musician groups perceived simultaneity of flash-tone stimuli over a narrower range of SOAs than the non-musician group. More interestingly, in assessing recalibration of flash-tone stimuli, the musician groups appeared to only be susceptible to auditory-leading adaptation while the non-musician group appeared to only be susceptible to visual-leading adaptation. This suggested that recalibration is a highly malleable process that may be influenced by different forms of expertise or experience. However, as noted by the authors, why musicians and non-musicians would differ in their susceptibility to recalibration remains unknown.

There is growing consensus that cumulative and rapid recalibration are independent mechanisms^8,28–30^, presumably to account for both short-term changes in the environment and long-term developmental changes in human physiology^28,31^. Some authors have suggested that rapid recalibration indexes a more sensory-driven process^8,16^ while cumulative recalibration predominantly reflects higher-order (decisional) processes^8,32^. Others have arrived at different or even opposite conclusions^33–36^. Since it is unclear why musicians and non-musicians would experience temporal recalibration differently, and given outstanding questions about how integration affects the two timescales of recalibration, we sought to expand upon Jicol and colleagues’^27^ findings and investigate how cumulative and rapid recalibration differed between musicians and non-musicians. Secondly, other studies typically describe their data using a mathematical function that has parameters that cannot distinguish between decisional and sensory components of the data (e.g., a Gaussian function)^37,38^. For example, while the standard deviation of the Gaussian is often assumed to reflect sensory precision, it is heavily influenced by task instructions that cause observers to adopt more or less liberal approaches to determining simultaneity^37^. Furthermore, the Gaussian function is unable to accurately capture common features of simultaneity judgement distributions, such as their asymmetry and their plateaued peaks where simultaneity is always perceived at several SOAs^39^. Therefore, we fit our data with an observer model developed by Yarrow and colleagues^40^. This provided a more formal investigation into how musicians and non-musicians integrate audiovisual temporal signals and whether any differences between the samples can be attributed to decisional or sensory processes.

## Methods

### Participants

Forty-eight participants volunteered for the experiment in exchange for course credit or A$15 per hour of participation. Six of these participants were excluded before analysis because survey data indicated that they did not meet the criteria for being a musician or a non-musician (see criteria below). Four additional participants (all non-musicians) were excluded because of poor fitting to the observer model (see details below). Hence, the final sample consisted of 20 musicians (*M*_age_ = 22.9, *SD* = 9.53, 14 females) and 18 non-musicians (*M*_age_ = 21.33, *SD* = 5.89, 13 females). Musicians and non-musicians did not significantly differ in age (*M*_diff_ = 1.57, *t*(36) = 0.60, *p* = 0.55), all participants reported that they did not have a current diagnosis of a vision or hearing impairment, and all except three reported being right-handed. Participants also took part in a spatial recalibration experiment in a separate session with the order of sessions counterbalanced across participants. The study was approved by the Queensland University of Technology (QUT) Human Research Ethics Committee (approval number 2000000530) and experiments conducted in accordance with the National Statement on Ethical Conduct in Human Research^41^. Written informed consent was obtained from participants.

Similar to many other studies using musicians^21,25,27^, we defined musicians as individuals with at least 6 years of practice playing their primary musical instrument and who had played this instrument for at least 1 h a week for the past 12 months. We defined non-musicians as individuals who had practiced playing any instrument for less than 12 months, excluding compulsory school lessons. For musician participants, we recorded the age at which they first started playing an instrument, the age at which they commenced formal music lessons, and primary instruments they played. For all participants, we also measured their scores on the Musical Training subscale on the Goldsmiths Musical Sophistication Index (GOLD-MSI) after the experiment^42^.

### Stimuli and apparatus

Participants were seated approximately 60 cm from a 23.8-inch computer monitor (Dell model P2419H, 60 Hz refresh rate, 1080p resolution) which displayed the visual stimuli. Auditory stimuli were presented binaurally through noise-cancelling headphones (Bose model QuietComfort 25). The visual stimuli consisted of a black fixation cross of 0.8° width and height and a flash (white annulus) with outer diameter 6° and inner diameter 4°, presented on a grey background. The auditory stimulus was a 2000 Hz tone presented at 65 dB (A), measured via a sound level meter (Protech model QM1589). Both the flash and the tone had a duration of 16.67 ms. Accuracy of stimulus duration and timing was confirmed via a slow-motion camera recording at 240 fps and video editing software. Responses were made on a computer keyboard, with participants using the “1” key to indicate a “synchronous” response and the “0” key to indicate an “asynchronous” response. The experimental displays were programmed using the Psychtoolbox extension for MATLAB R2020a^43,44^.

### Design and procedure

The experiment involved four phases: a baseline phase followed by a visual-leading adaptation phase, and a baseline phase followed by an auditory-leading adaptation phase. The order of the adaptation phases was counterbalanced across participants. Each baseline phase was a simultaneity judgement task conducted over two blocks. The adaptation phases followed a six-block procedure where each block consisted of ~ 2 min of adaptation immediately followed by a short post-adaptation simultaneity judgement task.

Each trial in the simultaneity judgement tasks began with the fixation cross and after a pseudorandom interval (800–1200 ms in 50 ms increments) the flash was presented. The tone was presented with one of nine SOAs relative to the flash (± 400, 266, 133, 66, and 0 ms; negative signs indicate the tone came first). The order of SOA conditions in the baseline blocks was completely random, whereas in the short post-adaptation blocks they were presented in randomly shuffled sets, where each set was comprised of each of the nine SOAs in a random order. This was to ensure that cumulative recalibration effects, which decay quickly^8^, were tested on all SOA conditions equally. After the offset of the last cue, there was a 500 ms interval before participants were prompted to respond with “synchronous” or “asynchronous”. In each phase, each SOA was presented 24 times for a total of 216 trials (i.e., 108 trials in each of the two blocks in the baseline tasks, and 36 trials in each of the six blocks for the post-adaptation tasks). This gave a total of 216 baseline trials and 216 post-adaptation trials per adaptation phase, providing 864 trials in total to assess rapid recalibration.

To minimise differences in response bias, at the start of the experiment we emphasised that participants should try to be as accurate as they can since the timing differences between the cues can be very small, but that ultimately they should respond with whatever they perceived. The experiment began with a short practice block where each SOA was presented three times in random order. Each adaptation block began with 120 presentations of a flash-tone stimulus with a constant SOA of either − 200 ms (auditory-leading) or 200 ms (visual-leading), depending on the phase. The interstimulus interval between each adaptation stimulus was pseudorandom (600–900 ms in 50 ms increments). Each adaptation block also had several catch trials (five on average) where the SOA was reversed. Specifically, there was a 5% chance on each trial (excluding the first and last 10 trials) that a catch trial would appear. Participants were told to press the spacebar as fast as they could whenever they perceived one of these catch trials. We used this catch trial procedure given evidence that it produces stronger recalibration effects than a simple probe detection task^45^.

### The observer model

**Figure 1:**
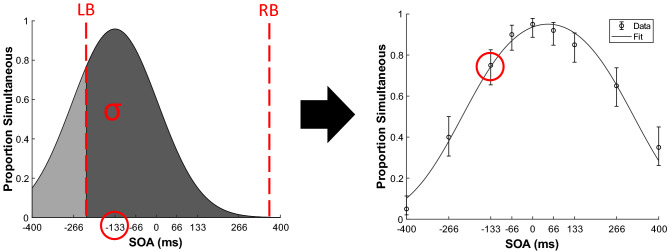
Visual depiction of the observer model and how the temporal integration window (TIW) is derived from it. The model assumes that when determining simultaneity, observers have a left boundary (LB) that determines whether the flash was simultaneous or late relative to the tone, and a right boundary (RB) that determines whether the tone was simultaneous or late relative to the flash. These are the first two parameters of the model (marked by red dashed lines in the left figure). Furthermore, on each trial the objective SOA (− 133 ms in this example) is assumed to be corrupted by Gaussian-distributed sensory noise, and therefore the *perceived* SOA will sometimes be greater or smaller than the *objective* SOA that was presented. The variance of this probability distribution of perceived SOAs (grey Gaussian distribution, σ) is the third and final parameter of the model. When the perceived SOA falls between the two boundaries, the model assumes that simultaneity is perceived. Hence, by calculating the area beneath the σ distribution where it lies between the two boundaries (the darker grey region in the left figure), we obtain the probability that the observer would perceive simultaneity at that SOA. These probabilities can be plotted as a psychometric function (the TIW). For example, the right figure shows the TIW obtained by fitting the model to a single observer’s data, with the example SOA circled in red and error bars denoting one standard error of the mean. By calculating the probability of perceiving simultaneity for each SOA and comparing these probabilities to the observed data, we thus compute the probability of obtaining the observed data given those parameter values. Then we use maximum likelihood estimation to find the values for the three parameters (LB, RB, and σ) that best fit the data.

To describe simultaneity perception, we fit an observer model (developed by Yarrow and colleagues^40^) to each participant’s data (see Fig. 1). This model assumes that two sensory cues are perceived as simultaneous when the difference in their perceived arrival times (i.e., their perceived SOA) falls between two decision boundaries. One of these boundaries is placed somewhere to the left of true simultaneity (i.e., deciding whether the sound preceded the flash or was presented at the same time as the flash) and the other is placed somewhere to the right of true simultaneity (i.e., deciding whether the sound was presented after the flash or at the same time as the flash). Crucially, each sensory cue’s arrival time is assumed to be subject to Gaussian-shaped sensory noise, meaning that the *difference* in their arrival times (perceived SOA) is also subject to Gaussian noise (with a variance equal to the sum of the variance of the two sensory cues). This measure of sensory noise is the final parameter of the model.

Hence, the model has three parameters, which are used to generate a psychometric function known as the temporal integration window (TIW). The first two parameters, the left and right decision boundaries, describe how liberal or conservative the observer is in determining simultaneity between two sensory cues. Visually, these boundaries mark each side of the psychometric function (and hence together they describe the width of the TIW). The final parameter represents sensory noise, which defines the slope of the two sides of the TIW. This parameter is measured by integrating the sensory noise distribution between the two boundaries and is therefore equivalent to the difference between two cumulative Gaussians (a more detailed description of the model can be found elsewhere^37^). A four-parameter version of this model has also been developed, which incorporates variability in the placement of the two decision boundaries^40^. We chose to use the simpler model as it provided a more parsimonious account of our data. Likelihood ratio tests between the two models revealed that the four-parameter model provided a significantly better fit on only 42 out of 252 fits, with the Akaike information criterion and the Bayes information criterion both providing the same interpretation.

### Analysis

Using the proportion of “synchronous” responses as a dependent variable, we fit an observer model to each participant’s judgements of whether a flash and a tone, presented at various SOAs, were simultaneous or not. We used MATLAB (version R2020a) for fitting the observer model, and the derived parameters of the model constituted dependent variables for statistical analyses (conducted using SPSS version 27). There were six fits to each participant’s data, one for each phase in the experiment and two for the rapid recalibration analysis (i.e., one fit for all trials in the experiment where the previous trial had an auditory-leading SOA, and one fit for trials where the previous trial had a visual-leading SOA). For each fit, we maximised the likelihood via the Nelder–Mead simplex algorithm with 245 starting points for each fit. Four participants (all non-musicians) were excluded from the analysis due to at least one of the six model fits being no better than a cumulative Gaussian fit (i.e., they were S-shaped, indicating that SOAs were not sampled widely enough on one side of the distribution).

Parameters of the models were then analysed by mixed analyses of variance (ANOVAs) with some or all of the following factors: musical training (musicians vs. non-musicians; between-subjects), phase (baseline vs. post-adaptation; within-subjects), modality order during adaptation (auditory-leading vs. visual-leading; within-subjects), and modality order of the preceding trial (auditory-leading vs. visual-leading; within-subjects). We had two primary interests: whether musical training modulated the magnitude of the model’s parameters (via the main effect of musical training), and whether musical training affected temporal recalibration (via interactions between musical training and the within-subjects factors).

## Results

### Participant characteristics

There are 7 items on the GOLD-MSI Musical Training subscale, and scores can range from 7 to 49. Musicians had a mean score of 36.95 (*SD* = 7.23) while non-musicians had a mean score of 9.11 (*SD* = 2.95), with this difference being statistically significant (*t*(36) = 7.68, *p* < 0.001, *d* = 4.95). The average age that musicians first started playing an instrument was 8.75 years (*SD* = 3.43), and of musicians who had received formal music lessons (all except one), the average age of onset was 8.58 years (*SD* = 2.39). The piano was the primary instrument for most of the participants (*n* = 7; see Supplementary Table S1 for the distribution of primary instruments in the sample).

While these data and the recruitment criteria for musician and non-musician participants helped establish a clear distinction between the groups, they did not allow for fine-grained differentiation of individual musician participants because they showed little variability within their group. For example, most musician participants had between 6 and 12 years of musical training experience due to their younger age. Thus, it was reasonable to examine the effects of musical training on temporal recalibration at a categorical level (i.e., musicians vs. non-musicians) instead of measuring them via continuous variables such as years of musical training.

### Group differences in sensory noise

**Figure 2:**
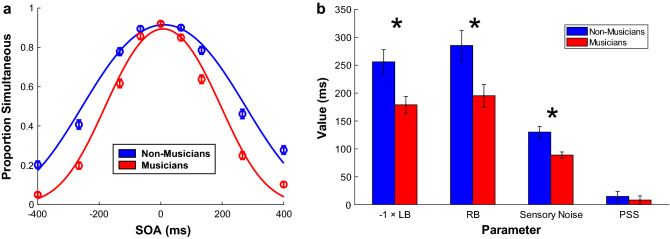
(**a**) The observer model fitted to simultaneity judgements for each group shows that musicians had a narrower temporal integration window (TIW) than non-musicians (i.e., musicians perceived synchrony between flashes and tones over a significantly narrower range of SOAs). Negative values indicate SOAs where the tone was presented first. (**b**) Musicians’ left boundary (LB; multiplied by − 1 in the figure) and right boundary (RB) were significantly closer to true simultaneity than non-musicians’, indicating they were stricter when integrating audiovisual cues. Musicians also had significantly reduced sensory noise, indicating greater sensory precision for audiovisual integration. The average of the boundaries (PSS) did not differ between the groups. Error bars show one standard error of the mean.

First, we assessed whether musicians and non-musicians differed in the precision with which they integrated the flash and the tone, as captured by the model’s sensory noise parameter (see Fig. 2). A mixed ANOVA with the between-subjects factor of musical training (musicians vs. non-musicians) and the within-subjects factors of phase (baseline vs. post-adaptation) and modality order during adaptation (auditory-leading vs. visual-leading) revealed a significant main effect of musical training (*F*(1, 36) = 12.65, *p* = 0.001, η^2^ = 0.22), such that the sensory noise parameter was smaller for musicians by 39.85 ms, indicating greater sensory precision when integrating audiovisual cues. There was also a significant main effect of phase (*F*(1, 36) = 14.03, *p* < 0.001, η^2^ = 0.03) and a significant interaction between modality order and musical training (*F*(1, 36) = 5.34, *p* = 0.03, η^2^ = 0.003). All other main effects and interactions were non-significant (*p*s > 0.07). The main effect of phase was caused by sensory noise being smaller in the post-adaptation phases than in the baseline phases (*M*_diff_ = − 13.26 ms). Post-hoc simple effects tests of the interaction (using a Bonferroni-adjusted alpha of 0.025) suggested that sensory noise was smaller during the auditory-leading phases than the visual-leading phases for non-musicians (*M*_diff_ = − 8.95 ms, *p* = 0.01) but not for musicians (*M*_diff_ = 0.96 ms, *p* = 0.75). This interaction is depicted in Supplementary Fig. S1. We then assessed whether sensory noise changed as a function of the modality order of the preceding trial (i.e., due to rapid recalibration). A mixed ANOVA with the between-subjects factor of musical training and the within-subjects factor of modality order (auditory-leading vs. visual-leading) revealed the expected main effect of musical training (*F*(1, 36) = 14.13, *p* < 0.001, η^2^ = 0.26) but found no main effect of modality order and no interaction (*p*s > 0.52). In summary, musicians exhibited reduced sensory noise relative to non-musicians, and sensory noise was reduced for both groups following adaptation.

### Cumulative and rapid recalibration effects

**Figure 3:**
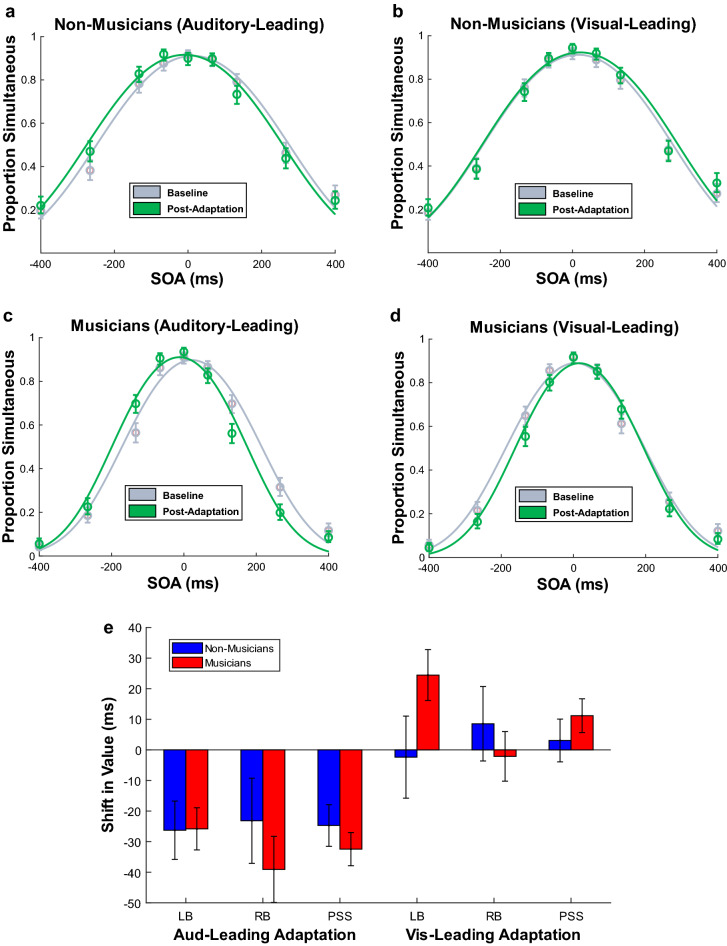
Group observer model fits depicting cumulative recalibration following auditory-leading and visual-leading adaptation. (**a**) Auditory-leading adaptation experienced by non-musicians. (**b**) Visual-leading adaptation experienced by non-musicians. (**c**) Auditory-leading adaptation experienced by musicians. (**d**) Visual-leading adaptation experienced by musicians. (**e**) Bar plot of parameter values showing that for both directions of cumulative recalibration, there were no significant group differences in the shift of the left boundary (LB), the right boundary (RB), or in the average of the boundaries (PSS). Furthermore, in both groups, only auditory-leading adaptation produced significant recalibration effects. Error bars show one standard error of the mean.

Cumulative recalibration was assessed by measuring the shift in the model’s decision boundaries from baseline to post-adaptation (see Fig. 3). We conducted two mixed ANOVAs (one for each decision boundary) with the between-subjects factor of musical training and within-subjects factors of phase and modality order during adaptation. For the left boundary, we found a significant main effect of musical training (*F*(1, 36) = 9.00, *p* = 0.005, η^2^ = 0.18) and a significant interaction between phase and modality order (*F*(1, 36) = 17.63, *p* < 0.001, η^2^ = 0.01). There was no interaction between musical training and modality order (*F*(1, 36) = 0.02, *p* = 0.88, η^2^ < 0.001), indicating that musical training did not modulate cumulative recalibration of the left boundary. All other effects were non-significant (*p*s > 0.14). The main effect of musical training was caused by musicians having a left boundary that was narrower (closer to 0 ms) by 76.53 ms, indicating a more conservative criterion for determining simultaneity. Planned simple effects tests for the interaction revealed that there was a significant shift in the left boundary from baseline to post-adaptation for auditory-leading adaptation towards auditory-leading SOAs (*M*_diff_ = − 26.05 ms, *F*(1, 36) = 20.16, *p* < 0.001, Hedges’ *g*_*av*_ = 0.28) but no significant effect of visual-leading adaptation (*M*_diff_ = 11.04 ms, *F*(1, 36) = 2.05, *p* = 0.16, Hedges’ *g*_*av*_ = 0.13). For the right boundary, we found a significant main effect of musical training (*F*(1, 36) = 7.20, *p* = 0.01, η^2^ = 0.15), a significant main effect of phase (*F*(1, 36) = 6.28, *p* = 0.02, η^2^ = 0.004), and a significant interaction between phase and modality order (*F*(1, 36) = 9.00, *p* = 0.01, η^2^ = 0.005). However, there was no interaction between musical training and modality order (*F*(1, 36) = 1.22, *p* = 0.28, η^2^ = 0.002), indicating that musical training did not modulate cumulative recalibration of the right boundary. All other effects were non-significant (*p*s > 0.24). The main effect of musical training was caused by musicians having a right boundary that was narrower by 90.27 ms, again indicating more conservative integration in musicians. The main effect of phase reflected an overall shift in the right boundary towards auditory-leading SOAs (− 14 ms). This effect, and the interaction, was caused by a significant shift in the right boundary from baseline to post-adaptation for auditory-leading adaptation towards auditory-leading SOAs (*M*_diff_ = − 31.13 ms, *F*(1, 36) = 12.75, *p* = 0.001, Hedges’ *g*_*av*_ = 0.27), with no significant effect of visual-leading adaptation (*M*_diff_ = 3.21 ms, *F*(1, 36) = 0.20, *p* = 0.66, Hedges’ *g*_*av*_ = 0.02). As depicted in Fig. 2, these results demonstrate that musicians have a far narrower TIW than non-musicians as they are more conservative when temporally integrating flash-tone cues. The left and right boundaries were both shifted towards auditory-leading SOAs following auditory-leading adaptation, but visual-leading adaptation had no effect on either boundary, and musical training did not modulate these effects (see Fig. 3).

**Figure 4:**
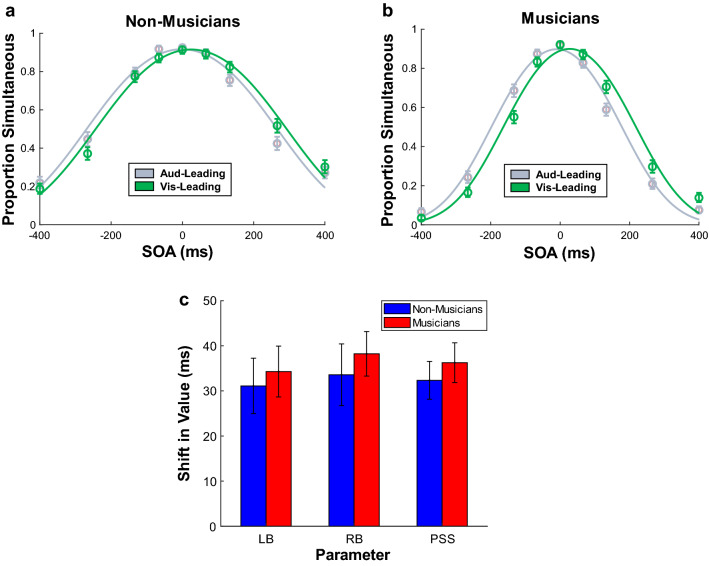
Group observer model fits for (**a**) non-musicians and (**b**) musicians show the rapid recalibration experienced by both groups (across all experimental phases), as denoted by a shift in the decision boundaries towards the modality order (auditory-leading or visual-leading) experienced on the previous trial. As shown by (**c**), there were no significant group differences in rapid recalibration of the left boundary (LB), the right boundary (RB), or the average of the boundaries (PSS). Error bars show one standard error of the mean.

We then assessed rapid recalibration (see Fig. 4). Rapid recalibration effects can persist across multiple trials, but the most sensitive way to assess rapid recalibration is by measuring the influence of each stimulus on the stimulus that follows it^9,14^. Therefore we calculated, across all trials in the experiment, how the modality order of the preceding stimulus shifted the left and right decision boundaries. We ran a mixed ANOVA for each boundary, with the between-subjects factor of musical training and the within-subjects factor of modality order (of the preceding trial; auditory-leading vs. visual-leading). For the left boundary, there were significant main effects of musical training (*M*_diff_ = 76.47 ms, *F*(1, 36) = 8.57, *p* = 0.01, η^2^ = 0.18) and modality order (*M*_diff_ = 32.70 ms, *F*(1, 36) = 61.70, *p* < 0.001, η^2^ = 0.03), but their interaction was not significant (*F*(1, 36) = 0.15, *p* = 0.70, η^2^ < 0.001). Similarly, for the right boundary there were significant main effects of musical training (*M*_diff_ = 93.12 ms, *F*(1, 36) = 7.62, *p* = 0.01, η^2^ = 0.17) and modality order (*M*_diff_ = 35.90 ms, *F*(1, 36) = 74.62, *p* < 0.001, η^2^ = 0.02), with no interaction (*F*(1, 36) = 0.31, *p* = 0.58, η^2^ < 0.001). The main effects of musical training were caused by musicians having narrower decision boundaries than non-musicians. The main effects of modality order demonstrated that rapid recalibration occurred for both boundaries, while the lack of an interaction between musical training and modality order indicated that musical training did not modulate rapid recalibration.

Finally, we assessed whether there was a relationship between sensory noise and each of our three types of recalibration: auditory-leading cumulative recalibration, visual-leading cumulative recalibration, and rapid recalibration. We tested this relationship on both decision boundaries separately, resulting in six linear regressions (using a Bonferroni-adjusted alpha of 0.0083). There were no significant relationships (*p* = 0.02 for visual-leading cumulative recalibration of the left boundary, and *p*s > 0.28 for the other five regressions; see Supplementary Fig. S2).

### Shifts in the point of subjective simultaneity (PSS)

Previous literature has typically measured recalibration in terms of the PSS (midpoint) of the TIW, so for ease of comparison to previous literature, we calculated this as the average of the two boundaries and ran the same analyses. A mixed ANOVA assessed shifts in the PSS due to cumulative recalibration. The PSS did not differ between musicians and non-musicians (*M*_diff_ = − 6.87 ms, *F*(1, 36) = 0.35, *p* = 0.56, η^2^ = 0.01) and musical training did not interact with phase (*F*(1, 36) = 0.001, *p* = 0.98, η^2^ < 0.001) or modality order during adaptation (*F*(1, 36) = 2.26, *p* = 0.14, η^2^ = 0.005)*.* However, there was a significant main effect of phase (*M*_diff_ = − 10.73 ms, *F*(1, 36) = 12.55, *p* = 0.001, η^2^ = 0.02) and modality order during adaptation (*M*_diff_ = 9.79 ms, *F*(1, 36) = 7.07, *p* = 0.01, η^2^ = 0.01), as well as an interaction between these two factors (*F*(1, 36) = 32.27, *p* < 0.001, η^2^ = 0.05). Planned simple effects tests of the interaction revealed the source of these effects: auditory-leading adaptation shifted the PSS towards auditory-leading SOAs (*M*_diff_ = − 28.59 ms, *p* < 0.001, Hedges’ *g*_*av*_ = 0.73) but visual-leading adaptation did not shift the PSS (*M*_diff_ = 7.13 ms, *p* = 0.12, Hedges’ *g*_*av*_ = 0.18). A second mixed ANOVA assessed shifts in the PSS due to rapid recalibration. There was a significant main effect of modality order (indicating that the PSS shifted due to rapid recalibration; *M*_diff_ = 34.30 ms, *F*(1, 36) = 125.72, *p* < 0.001, η^2^ = 0.18) but there was no main effect of musical training (*M*_diff_ = − 8.33 ms, *F*(1, 36) = 0.50, *p* = 0.49, η^2^ = 0.01) and there was no interaction between musical training and modality order (*F*(1, 36) = 0.41, *p* = 0.53, η^2^ < 0.001). Thus, for both timescales of recalibration, we arrived at the same conclusions irrespective of whether we analysed shifts in the decision boundaries or in the PSS (see Figs. 2, 3, 4).

## Discussion

Our results support a growing collection of evidence^21–23,25,27^ that musicians have more precise audiovisual integration in the temporal domain. Specifically, musicians integrated audiovisual cues over a narrower range of SOAs than non-musicians due to having more conservative criteria *and* reduced sensory noise when making simultaneity judgements. As our study used simple flash-tone stimuli, we have provided further evidence that refined temporal integration in musicians is a domain-general phenomenon and is not restricted to musical or speech stimuli. On the other hand, while both cumulative and rapid recalibration effects were observed in both groups, musical training did not modulate any form of recalibration, despite previous suggestions^27^. Surprisingly, only auditory-leading adaptation produced cumulative recalibration in our sample, with little to no effect of visual-leading adaptation on the boundaries of the temporal integration window (TIW).

Via an observer model, we found that musicians had narrower (more conservative) decision boundaries while also exhibiting reduced sensory noise. Note that there is an inherent ambiguity in determining the specific neural processes that underpin the model’s decisional and sensory parameters. For example, the sensory noise parameter in the model could represent neural noise in terms of latency, spike rates, or some other process that does not necessarily represent an early stage of sensory perception, and it is similarly unclear which mechanisms drive the placement of the model’s decision boundaries^40^. Moreover, both of these parameters could be modulated by cognitive factors, and there is a broad literature assessing differences in cognitive functions between musicians and non-musicians. Some studies have found that musical training enhances cognitive functions such as selective attention and working memory^46,47^, which may modulate the extent to which observers integrate multisensory stimuli^48,49^.

The structural differences underlying refined temporal integration in musicians require further investigation. It is possible that musical training refines multisensory integration via enhancements in unimodal regions of the brain. For example, the auditory cortex receives multisensory thalamic inputs^20^ and musicians have exhibited greater cortical volume in the auditory cortex^50^. Musicians may also process multisensory information differently within the auditory cortex^51^. Furthermore, since playing an instrument presumably activates several sensory cortices at once, it is possible that musical training strengthens the connections between sensory regions via Hebbian-like mechanisms^20^. Consistent with this idea, musicians have been found to have stronger white matter connections between the auditory cortex and anterior regions of the brain^52^, as well as greater multisensory facilitation of reaction times to audiotactile stimuli^24^.

An alternative possibility is that musicianship-related enhancements are specific to multisensory structures of the brain rather than unisensory regions and the connections between them^53^. MEG data has shown that musicians respond more strongly to asynchronous stimuli in the left cerebellum and more strongly to synchronous stimuli in various regions in the temporal lobe including the superior temporal gyrus, a multisensory region^23^. Musicians also show greater neural activity in response to asynchronous musical cues and greater neural connectivity in response to music in a circuit involving the superior temporal sulcus, premotor cortex, and cerebellum^21,22^. Finally, fMRI data has shown that brain activity in the cerebellum and other regions is reduced in drummers (relative to non-musicians) when viewing synchronised drumming actions, possibly reflecting greater neural efficiency^54^.

Lee and Noppeney^21^ provide a more specific theory about the mechanisms underlying musicians’ refined multisensory integration. They argue that musical training might refine audiovisual integration via predictive coding mechanisms, whereby predictions about motor actions and their sensory consequences are continually refined via feedforward and feedback neural connections. Indeed, the involvement of the motor system in producing music and in integrating sensory cues seems special in conferring multisensory perceptual benefits. Lappe et al.^55,56^ trained participants for 2 weeks in either playing a piano piece (sensorimotor-auditory training) or auditorily evaluating how accurately the other group performed these piano pieces (auditory-only training). Compared to the auditory-only training group, the sensorimotor-auditory group exhibited enhanced MEG responses (mismatch negativity) to melodic and rhythmic musical deviants. One prominent theory of timing perception is that human observers calibrate multisensory timing through motor actions that produce sensory consequences^3^. Since playing an instrument is a unique activity where precise motor actions produce many specific and predictable auditory consequences, this may directly account for musicians’ refined audiovisual simultaneity perception.

Irrespective of the underlying mechanisms, our data provide evidence that the precision with which an observer integrates audiovisual signals does not predict the magnitude with which their brain compensates for misalignment between the signals (i.e., recalibration). Despite earlier reports that narrower TIWs were associated with reduced rapid recalibration^9–11^, later studies have shown that these relationships were likely accounted for by age^57^. Furthermore, a recent study where participants made temporal order judgements found that rapid recalibration effects were predicted by the physical, rather than the perceived, temporal order of the preceding audiovisual cues^16^. These results may suggest that the brain updates its perception of multisensory timing based on how physically misaligned the sensory cues are, rather than whether these cues were perceived as a unitary signal. As argued by Van der Burg et al.^16^, such a process could be beneficial in avoiding the potential consequences of a decisional drift whereby erroneous judgements of simultaneity become larger and larger in a snowball-like fashion (for example, once perceived simultaneity is pulled in one direction, it becomes more difficult for it to be pulled in the other direction). This remains surprising given the intuitive assumption that recalibration should be stronger when two asynchronous sensory cues are perceived to be related to the same event. Hence, the relationship between multisensory integration and recalibration should be explored further, perhaps by utilising different measures of integration (e.g., the redundant target effect^58^).

The finding that musical training did not modulate temporal recalibration may suggest there is a dissociation between recalibration and the precision with which observers integrate audiovisual cues, at least in the temporal domain. Such a notion is consistent with the absence of significant relationships between sensory noise and recalibration in our data. However, a previous study found that musical training did modulate temporal recalibration, as musicians were only susceptible to auditory-leading flash-tone adaptation while non-musicians were only susceptible to visual-leading flash-tone adaptation^27^. By contrast, if we were to find anything suggestive of musicianship-related modulation of recalibration in our study, it would be that musicians might have shown some (statistically unsubstantiated) shift of the left boundary in *visual*-leading adaptation while non-musicians clearly did not (see Fig. 3c). Perhaps these conflicting findings could be attributed to the different techniques used to model the data (i.e., Gaussian fitting^27^ versus using an observer model), or to differences in experimental design. For example, we designed our study so that cumulative and rapid recalibration could be measured independently, with multiple short post-adaptation blocks, whereas Jicol and colleagues^27^ used a single long post-adaptation block, maintaining recalibration by intermittently presenting adaptation stimuli throughout this block. It is also possible that the musician samples in each study differed in important characteristics, such as the age when they first started playing music, the different instruments they play, and how often they play. Finally, we cannot rule out the possibility that our null findings were due to insufficient power as we did not conduct an a priori power analysis, instead taking a heuristics-based approach and aiming for a sample size equal to or greater than previous studies in the field^21–27^. However, this seems unlikely given that we observed extremely small effect sizes when assessing whether musical training interacted with cumulative (left boundary η^2^ = 3.22 × 10^–5^, right boundary η^2^ = 1.84 × 10^–3^) and rapid recalibration (left boundary η^2^ = 1.29 × 10^–4^ and right boundary η^2^ = 3.91 × 10^–5^).

Although we observed typical cumulative recalibration for auditory-leading adaptation—that is, a shift in perception of subjective simultaneity towards auditory-leading test stimuli (via the left and right boundaries of the TIW)—visual-leading adaptation had no reliable effect on the boundaries of the TIW. This could be considered surprising since the visual-leading side of the TIW is typically wider than the auditory-leading side and has greater training- and expertise-driven plasticity^22,59^. However, asymmetry in recalibration effects is highly plausible when considering differences in the physical and neural transmission speeds of sound and light^1^. For example, sound becomes infinitely delayed relative to light as the physical distance between the observer and the stimulus increases; where physical distance is negligible, neural transmission^2^ and perceptual phenomena like the prior entry effect^60^ would predict sound to precede light only by about 50 ms. Hence, an adaptation asynchrony of − 200 ms (sound first) is far more unusual than an asynchrony of 200 ms (light first). It is therefore possible that auditory-leading adaptation produces stronger recalibration effects because it sends a stronger signal to the brain that its perception of multisensory timing needs updating. This conclusion seems at odds with behavioural evidence that rapid recalibration is driven by visual-leading asynchronies^9,34^, but it is consistent with electroencephalographic (EEG) data showing that rapid recalibration is more robust when the *current* trial has an auditory-leading asynchrony^34^. Our results suggest that future studies should investigate differences in how the two sides of the TIW are recalibrated due to audiovisual asynchrony.

In conclusion, we found that musical training refines audiovisual integration in the temporal domain but does not affect how the brain shifts its perception of audiovisual timing after exposure to asynchronous audiovisual cues (temporal recalibration). This supports the notion that there is a dissociation between temporal recalibration and perceptual integration of multisensory stimuli. Future research should continue to explore how individual differences and prior experiences affect multisensory integration and recalibration, and the extent to which integration and recalibration are dependent upon each other.

## Supplementary Information

Supplementary Information.

## Data Availability

Data and code for stimulus presentation and analysis are available on the Open Science Framework: https://osf.io/4hyt2/.
