# Supplementary material for: Musical training refines audiovisual integration but does not influence temporal recalibration
Source: Sci Rep. 2022 Sep 12;12:15292. doi: 10.1038/s41598-022-19665-9 (PMC9468170; doi:10.1038/s41598-022-19665-9)
Supplement: Supplementary file 1 — Supplementary Information. [file 41598_2022_19665_MOESM1_ESM.docx]

**Musical training refines audiovisual integration but does not influence temporal recalibration.**

Matthew O’Donohue^1*^, Philippe Lacherez^1^, and Naohide Yamamoto^1^

^1^Queensland University of Technology (QUT), School of Psychology and Counselling, Kelvin Grove, QLD 4059, Australia

^*^mp.odonohue@qut.edu.au

**Supplementary Material**

| **Primary Instrument(s)** | **Frequency** |
| --- | --- |
| Clarinet | 1 |
| Piano | 6 |
| Drums | 1 |
| Guitar/Bass | 4 |
| Violin | 2 |
| Cello | 1 |
| Saxophone | 4 |
| Piano/Flute/Alto Saxophone* | 1 |

* This category is for one participant who listed multiple primary instruments.

**Table S1.** Distribution of primary instruments in the musician sample.


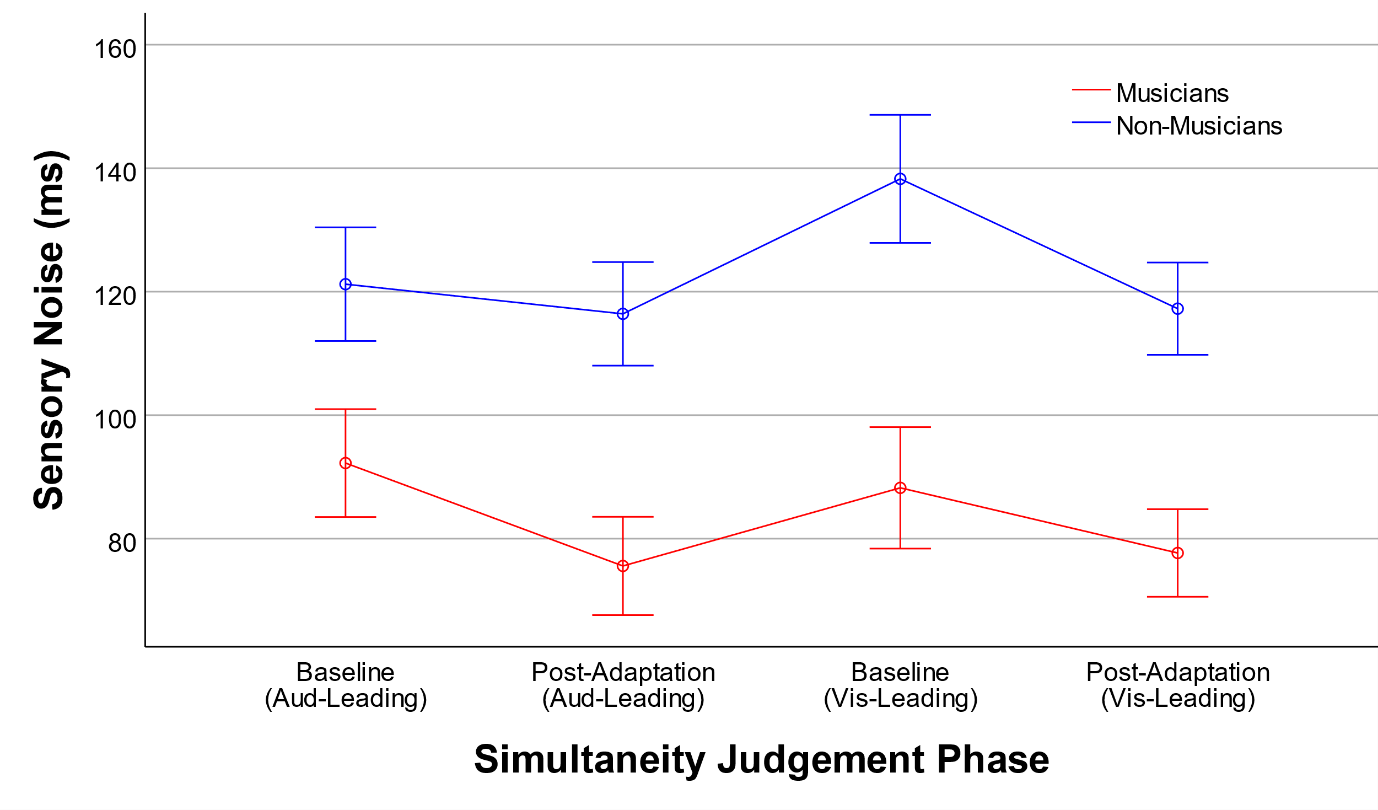


**Figure S1.** Sensory noise as a function of each phase in the experiment (see main text for statistical tests). Sensory noise was smaller in the post-adaptation phases than in the baseline phases. There was a significant interaction between adaptation and musical training, where sensory noise was smaller in the auditory-leading adaptation phases than in the visual-leading adaptation phases, but for non-musicians only. The plot suggests that this was caused by sensory noise being unusually high in the visual-leading adaptation baseline phase for non-musicians. Since the two types of adaptation were counterbalanced, it is unclear why this occurred. Error bars show one standard error of the mean.


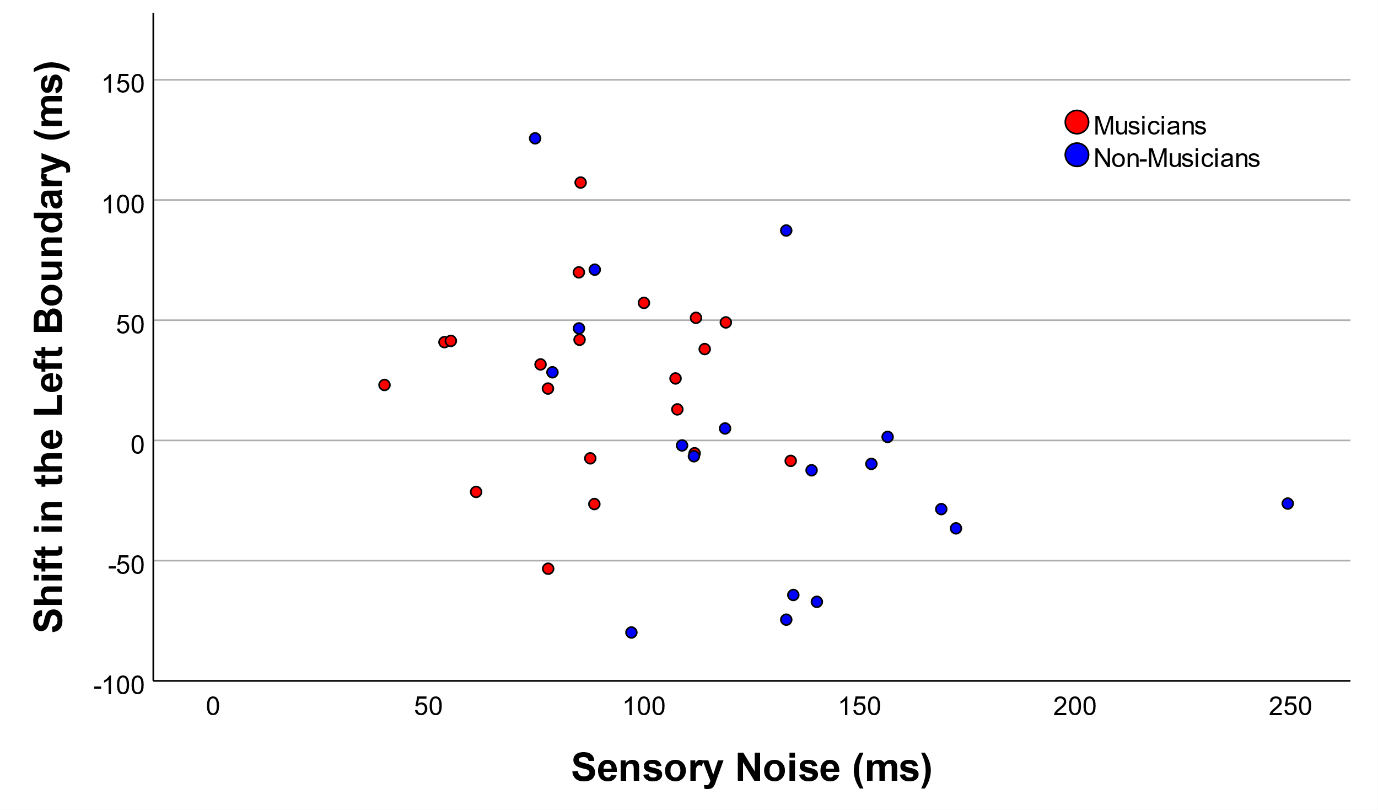


**Figure S2.** This scatterplot shows the non-significant relationship (*R*^2^ = .15, *b* = -.48, *F*(1, 36) = 6.51, *p* = .02) between sensory noise and the shift in the left boundary following visual-leading adaptation. Red and blue data points represent musicians and non-musicians, respectively. The non-significant trend is that as sensory noise decreased, visual-leading cumulative recalibration of the left boundary became stronger. Note that musical training did not account for the non-significance of this relationship, as the regression was non-significant for both musicians (*R*^2^ < .001, *b* = .02, *F*(1, 18) = .003, *p* = .96) and non-musicians (*R*^2^ = .19, *b* = -.58, *F*(1, 16) = 3.69, *p* = .07). Furthermore, when other studies have observed a trend between integration and recalibration, it is in the opposite direction to what is being shown here (e.g., Van der Burg et al., 2013; see main text). We conducted five other linear regressions between sensory noise and recalibration and all of these were also non-significant (*p*s > .28).
